# The changing pattern of human brucellosis: clinical manifestations, epidemiology, and treatment outcomes over three decades in Georgia

**DOI:** 10.1186/1471-2334-10-346

**Published:** 2010-12-09

**Authors:** Tamar Akhvlediani, Danielle V Clark, Giulen Chubabria, Otar Zenaishvili, Matthew J Hepburn

**Affiliations:** 1 Clinical Research Unit, Technology Management Company, Tbilisi, Georgia; 2 Walter Reed Army Institute of Research, Silver Spring, MD, USA; 3 Institute of Parasitology and Tropical Medicine, Tbilisi, Georgia; 4 United States Army Medical Research Institute of Infectious Diseases, Frederick, MD, USA

## Abstract

**Background:**

Brucellosis is an endemic infection in Georgia. We conducted a review of patient records with a suspected or confirmed diagnosis of brucellosis over three decades at the central referral hospital for brucellosis cases, the Institute of Parasitology and Tropical Medicine (IPTM) in Tbilisi. The purpose was to describe the demographic profile and clinical characteristics as well as diagnostic and treatment strategies in patients with brucellosis.

**Methods:**

Data were abstracted from randomly selected patient records at the IPTM. In total, 300 records were reviewed from three time periods: 1970-73, 1988-89, and 2004-2008.

**Results:**

The age distribution of patients shifted from a median age of 40 years in the first time period to 20 years in the third time period. Azeri ethnicity was an increasing proportion of the total number of cases. The frequency of relapsed infection was 14.7% (44 cases). A total of 50 patients received vaccine therapy, and although the vaccine produced immune responses, demonstrated by an increase in agglutination titers, it was not associated with improved outcome.

**Conclusion:**

The demographics of brucellosis in Georgia fit a profile of persons that tend sheep. Osteoarticular complications were commonly detected, especially in children. The changing pattern of brucellosis in Georgia suggests clinicians should be updated about different trends in brucellosis in their country.

## Background

Brucellosis is one of the most common zoonotic infections, with more than 500,000 new cases reported annually worldwide [1]. The majority of cases occur in the Mediterranean countries of Europe and Africa, Middle East, India, Central Asia, Mexico, and Central and South America [2]. Before an etiology was determined, brucellosis was known according to endemic geographical areas: Mediterranean fever, Gibraltar fever, Malta fever, Cyprus fever, and Danube fever. Most experts believe the number is only a small fraction of the overall annual incidence [3]. Although the routes of transmission have been known for decades, and campaigns for eradication have been attempted in many countries, the consistently high number of cases reflects the challenges associated with preventing the transmission of this pathogen.

Brucellosis is caused by infection with *Brucella *species bacteria. The organism is a pleomorphic, gram-negative, non-spore-forming coccobacillus. They are facultative intracellular pathogens, localized predominantly in organs with numerous macrophages such as lung, spleen, liver, bone marrow, and synovium. Presently, there are six recognized species of *Brucella*, four of which are pathogenic to humans: *B melitensis, B, abortus, B. suis*, and *B. canis *[4].

Human infection can occur through consumption of contaminated, unpasteurized animal products, direct contact with infected animal parts, and through the inhalation of infected aerosolized particles. Brucellosis is an occupational disease in shepherds, abattoir workers, veterinarians, dairy-industry professionals, and personnel in microbiology laboratories [5,6]. Transmission of *B. melitensis *from person-to-person has also been reported in the literature [7-9].

Human brucellosis is a systemic infection that may manifest with a myriad of non-specific symptoms (e.g., fever, sweats, malaise, anorexia, headache, back pain) as well as substantial residual disability. The onset can be insidious or acute, generally beginning within 2 to 4 weeks after inoculation. An "undulant" fever pattern is apparent in patients who are untreated for long periods of time [10]. Osteoarticular disease is the most common complication, followed by the involvement of reproductive system. Endocarditis remains the principal cause of mortality in the course of brucellosis [11]. Childhood brucellosis generally exhibits a more benign course in terms of the rate and severity of complications and the response to treatment [12,13].

Brucellosis is an endemic infection in Georgia. According to the data of the National Center of Disease Control and Public Health of Georgia, 168 and 175 cases of brucellosis were reported in 2008 and 2009, respectively. However, the real numbers can be higher because not all the patients seek medical treatment and even if they do, not all the cases are reported by the physicians to the public health system. The brucellosis is diagnosed based on the slide (Huddelson) and tube (Right) agglutination tests. The treatment implies traditional three course treatment with antibiotics discussed below. However, WHO recommended treatment is becoming more popular among physicians. The first case of brucellosis in Georgia, with serological and microbiological confirmation, was described by Dr. N. Makhviladze in 1923 [14]. In the following decades, brucellosis was the subject of extensive observation. Most suspected brucellosis patients in Georgia are referred to the Institute of Parasitology and Tropical Medicine (IPTM) in Tbilisi. It is estimated that the IPTM annually tests about 400 clinical samples for serological evidence of brucellosis, with approximately 150 positive tests reported per year. The diagnosis of brucellosis in Georgia has always been based on the slide and tube agglutination tests, and has not changed over three decades.

Health care has changed substantially in Georgia in the last 40 years, due to advances in science and medicine as well as social and political upheaval and reform. In the period before the collapse of the Soviet Union, subsidized medical care resulted in minimal costs for patients, thereby encouraging utilization of medical services. Currently many health services are becoming privatized, and health insurance is not widely available. However, diagnostics and treatment for brucellosis remain subsidized by the Georgian government at the IPTM.

In the 1970s, vaccine therapy was administered either in conjunction with antibiotics or as a separate therapeutic option. Heat-inactivated *Brucella *polyvalent vaccine was injected intravenously to activate the immune system, which was regarded as a curative reaction [15]. Vaccine therapy was mainly indicated for chronic brucellosis. However, it was occasionally used in acute cases, at the discretion of the treating physician [16].

Examination of the epidemiology and clinical characteristics of this infection was not systemically conducted in the recent past in Georgia. It is important that health care providers and public health leaders are aware of current trends in brucellosis, because the scenarios in which brucellosis is acquired, as well as the types of patients exposed and duration of infection before presentation, are influenced by multiple factors and may change over time. Assumptions about brucellosis from 10-20 years ago may be incorrect, leading to inaccurate clinical suspicion of infection and inappropriately targeted public health surveillance efforts.

To address the lack of recent data on human brucellosis in the region and explore changes in the epidemiology and clinical presentation and course over time, we conducted a medical chart review of suspected or confirmed brucellosis patients at the IPTM in Tbilisi. The purpose of the study was to better understand the demographic distribution as well as clinical manifestations and the patterns of response to treatment of the disease. The review allowed for comparisons in management strategies over three decades.

## Methods

Data were abstracted from randomly selected inpatient records of suspected or confirmed brucellosis patients at the IPTM. Before initiation, this study was reviewed by the Institutional Review Board (IRB) and it was determined not to constitute the research involving human subject. An exemption certificate attesting this fact was issued by the IRB.

In total, 300 records were reviewed from three time periods: 1970-73, 1988-89, and 2004-2008. Patients who were initially suspected to have brucellosis but were then given an alternate final diagnosis were excluded from most analyses. The first time period was chosen to characterize a period where vaccine therapy was still used; the second is a period before the collapse of the Soviet Union, when subsidized medical care resulted in minimal costs for patients, thereby encouraging utilization of medical services. The most recent time period was selected to evaluate the current situation of referral patterns for brucellosis. The charts were reviewed by a single investigator which ensured uniformity in the data recording. Information collected included demographic information, risk factors for potential exposure to brucellosis, clinical manifestations, diagnostic laboratory tests, and prescribed treatment regimens. Documentation of relapsed infection was also recorded.

Demographic, epidemiologic, and clinical parameters were assessed using chi-squared and Fisher exact tests. A p-value less than 0.05 was considered significant. Logistic regression was used to further evaluate associations detected in bivariate analysis. All analyses were conducted using Epi Info™Version 3.4 (Centers for Disease Control and Prevention, Atlanta, GA).

## Results

### Epidemiologic changes over time

**Figure 1:**
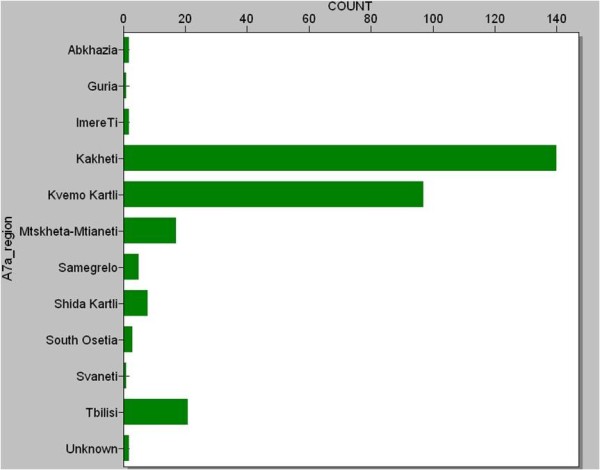
**Frequency of Brucellosis Cases in Regions of Georgia**.

Most brucellosis patients (87.6%) were from eastern Georgia, with the most common regions for brucellosis cases being the Kvemo Kartli and Kakheti regions (figure 1). Traditionally, animal exposures, especially sheep husbandry, were more prevalent in eastern Georgia. In the first time period, there were more cases in Kvemo Kartli region, while Kakheti predominated in the second and third time points. Shepherd (29%) was the most common occupation followed by farmer (12.3%).

Azeri ethnicity was an increasing proportion in the total number of cases. By the most recent time, 62% of the patients were Azeris (Table 1), which represent only 6.5% of the Georgian population. The prevalence of brucellosis was much higher in men (85%). The age distribution of patients shifted from a median age of 40 years in the first time period to 20 years in the third time period. There were 11 children less than 10 years with brucellosis (9 of them in the third time period) and 54 between 10 and 20 years (39 in the third time period).

**Table 1:** Demographic Characteristics of Brucellosis Patients by Decade

Demographic Group	Decade 1	Decade 2	Decade 3
**Gender**			

**Male**	66 (77.6%)	79 (88.8%)	89 (89%)

**Female**	19 (22.4%)	10 (11.2%)	11 (11%)

**Age Group**			

**1-9**	2 (2.4%)	0	9 (9.1%)

**10-19**	4 (4.8%)	11 (12.5%)	39 (39.4%)

**20-29**	17 (20.2%)	32 (36.4%)	23 (23.2%)

**30-39**	23 (27.4%)	15 (17%)	14 (14.1%)

**40-49**	18 (21.4%)	4 (4.5%)	6 (6.1%)

**50-59**	14 (16.7%)	21 (23.9%)	6 (6.1%)

**≥60**	6 (7%)	5 (5.7%)	2 (2.0%)

**Nationality**			

**Georgian**	37 (43.5%)	48 (53.9%)	36 (36%)

**Azerbaijani**	19 (22.4%)	33 (37.1%)	62 (62%)

**Armenian**	14 (16.5%)	5 (5.6%)	0

**Other**	15 (17.6%)	3 (3.4%)	2 (2%)

**Occupation**			

**Farmer**	10 (11.8%)	10 (11.2%)	12 (12%)

**Shepherd**	19 (22.4%)	31 (34.8%)	30 (30%)

**Housewife**	7 (8.2%)	6 (6.7%)	7 (7%)

**Student**	2 (2.4%)	5 (5.6%)	12 (12%)

**Retired**	7 (8.2%)	4 (4.5%)	1 (1%)

**Other**	40 (47.1%)	33 (37.1%)	38 (38%)

**Total**	85	89	100

An accurate description of potential risk factors for developing brucellosis was not available in some of the medical charts. In cases of relapsed or latent brucellosis, the epidemiological information was not provided, assuming that it was collected when the patient presented with acute infection. A large majority (86.3%) of patients had contact with animals before symptom development. In 12.7%, animal contact was the only type of exposure. A total of 40.4% of patient records noted contact with sheep, 35.0% had contact with both sheep and cattle. Consumption of unpasteurized products was noted in 68.0% of patients, with 16.5% of cases having exposure solely with animal products (Table 2). One patient had an exposure to a live attenuated vaccine during animal vaccination.

**Table 2:** Exposure Types in Patients with Brucellosis

Exposure	Frequency	Percent
**Contact with animal**	27	12.7%

**Contact with animal product**	35	16.5%

**Contact with both animal and animal product**	148	69.8%

**Vaccine**	1	0.5%

**Other**	1	0.5%

**Total**	212	100.0%

### Diagnosis of brucellosis

The core criteria used to diagnose patients with brucellosis did not change over time. In all three time periods, diagnosis of brucellosis was based on the Wright and Huddelson agglutination tests, in combination with clinical and epidemiological information. All patients were tested at least once for agglutinating antibodies, and 50% of patients were tested twice. The tube agglutination titer of 1:200 and higher was considered positive for brucellosis. Cultures of blood or other tissue samples were not routinely performed in any time period. An additional diagnostic test practiced mostly in the first time period is an allergy skin test. The Burnè skin test was conducted using Melitin (culture filtrate, toxin in broth). In total, 66 patients were tested with the Burnè skin test (64 in the first and 2 in the second time period) and 44 were positive. This test is no longer used because of the high rate of side effects, particularly severe allergic reactions [14,17]. The laboratory analyses consistently included complete blood count (CBC), urinalysis, C-reactive protein (CRP), erythrocyte sedimentation rate (ESR), and blood glucose.

Patients were categorized as brucellosis primaria (primary or acute brucellosis), brucellosis latenta (subacute course of infection), brucellosis recidiva (relapsed brucellosis), and status post-brucellosis (residual/chronic disease). Criteria for categorization are somewhat arbitrary, which is consistent with observations for other publications [11]. The final diagnosis was brucellosis in 91.6% of charts. Primary brucellosis was the diagnosis in 52.5% of presenting patients. Alternative diagnoses included polyarthritis (30.8%) and chronic cholecystitis (26.9%).

### Clinical Characteristics

Patients with a final diagnosis of brucellosis were 2.9 times more likely to have documented fever on admission than non-brucellosis patients (p = 0.02). No other symptoms were significantly different between brucellosis and non-brucellosis patients. The remaining analyses pertain only to patients with a final diagnosis of brucellosis. Fever, arthralgias, and sweats were the most frequently recorded symptoms for acute brucellosis patients (Table 3). Among relapse, latent, and chronic brucellosis patients, arthralgia was the most frequently noted symptom. Neuropsychiatric symptoms such as depression, difficulty concentrating and sleep disturbance, were observed rarely. The clinical symptoms did not differ over time.

**Table 3:** Frequency of Clinical Symptoms on Admission

Symptom*	Acute N (%)	Relapsed N (%)	Latent N (%)	Chronic N (%)	Total N (%)
**Fever**	129 (82.2)	31 (70.5)	41 (66.1)	3 (30.0%)	204 (74.7)

**Sweats**	112 (71.3)	24 (54.5)	35 (56.6)	4 (40.0)	175 (64.1)

**Rigors**	20 (12.8)	6 (13.6)	5 (8.1)	1 (10.0)	32 (11.8)

**Malaise**	81 (51.6)	19 (43.2)	29 (46.8)	1 (10.0)	130 (47.6)

**Fatigue**	63 (40.1)	9 (20.5)	16 (25.8)	1 (10.0)	89 (32.6)

**Aches**	28 (18.2)	17 (40.5)	20 (32.3)	2 (20.0)	67 (25.0)

**Arthralgia**	138 (87.9)	37 (84.1)	55 (88.7)	9 (90.0)	239 (87.5)

**Arthritis**	20 (12.7)	5 (11.4)	7 (11.3)	1 (10.0)	33 (12.1)

**Myalgia**	17 (10.8)	5 (11.4)	3 (4.8)	1 (10.0)	26 (9.5)

**Bursitis**	1 (0.6)	0	4 (6.5)	0	5 (1.8)

**Anorexia**	10 (6.4)	2 (4.5)	1 (1.6)	0	13 (4.8)

**Abdominal Pain**	8 (5.1)	3 (6.8)	5 (8.1)	1 (10.0)	17 (6.3)

**Hepatomegaly**	38 (24.2)	15 (34.1)	14 (22.6)	2 (20.0)	69 (25.3)

**Splenomegaly**	14 (8.9)	5 (11.4)	2 (3.2)	0	21 (7.7)

**Lymphadenopathy**	15 (9.6)	7 (15.9)	4 (6.5)	0	26 (9.5)

**Cough**	4 (2.5)	1 (2.3)	4 (6.5)	0	9 (3.3)

Testicular Pain**	12 (9.0)	4 (10.0)	0	0	16 (6.8)

Testicular swelling**	14 (10.5)	3 (7.5)	0	0	17 (7.3)

**Total**	157	44	63	10	274

*The following symptoms were assessed in the medical charts, but were recorded in fewer than five charts and are therefore not listed in the table above: weight loss, diarrhea, constipation, jaundice, sore throat, tonsillitis, pneumonia, depression, difficulty concentrating, skin lesions, and ocular symptoms.

**Percentages reflect only male patients

Pediatric records were significantly more likely to note fever on admission (OR = 3.7, p = 0.02) and less likely to report aches (OR = 0.16, p = 0.0004). The maximum temperature recorded was significantly higher in children (38.6°C versus 38.2°C, p = 0.02) controlling for the classification of brucellosis (i.e., acute, relapse, latent, or chronic). Pediatric records were also 2.8 times more likely to note arthritis controlling for decade and classification of brucellosis (p = 0.05) (Table 4).

**Table 4:** Comparison of the clinical presentations of acute brucellosis in adult and pediatric patients

Symptom*	Pediatric N (%)	Adult N (%)	Total N (%)
**Fever**	30 (88.2)	98 (81.0)	128 (82.6)

**Sweats**	27 (79.4)	83 (68.6)	110 (71.0)

**Rigors**	5 (14.7)	15 (12.5)	20 (13.0)

**Malaise**	20 (58.8)	59 (48.8)	79 (51.0)

**Fatigue**	17 (50.0)	46 (38.0)	63 (40.6)

**Aches**	2 (5.9)	25 (21.2)	27 (17.8)

**Arthralgia**	32 (91.1)	104 (86.0)	136 (87.7)

**Arthritis**	10 (29.4)	9 (7.4)	19 (12.3)

**Myalgia**	2 (5.9)	15 (12.4)	17 (11.0)

**Bursitis**	0	1 (0.8)	1 (0.6)

**Anorexia**	4 (11.8)	6 (5.0)	10 (6.5)

**Abdominal Pain**	0	8 (6.7)	8 (5.2)

**Hepatomegaly**	10 (29.4)	27 (22.3)	37 (23.9)

**Splenomegaly**	5 (14.7)	9 (7.4)	14 (9.0)

**Lymphadenopathy**	8 (23.5)	7 (5.8)	15 (9.7)

**Cough**	1 (2.9)	3 (2.5)	4 (2.6)

Testicular Pain**	1 (2.9)	11 (9.1)	12 (7.7)

Testicular swelling**	1 (2.9)	13 (10.7)	14 (9.0)

**Total**	34	121	155

*The following symptoms were assessed in the medical charts, but were recorded in fewer than five charts and are therefore not listed in the table above: bursitis, weight loss, diarrhea, constipation, jaundice, sore throat, tonsillitis, pneumonia, depression, difficulty concentrating, skin lesions, and ocular symptoms.

**Percentages reflect only male patients

Forty-eight patients presented with complicated disease, including osteoarticular complications (32), orchitis (15), and hepatitis (1). There were significantly more complications recorded over time; there were seven complicated cases in the first decade, as compared to 15 and 27 in the second and third time periods, respectively (p = 0.004). People with complications did not have rising agglutination titers, and did not have a higher initial titer on average. There were 44 brucellosis patients with relapsed infection (16%), with the majority of relapse cases recorded in the first time period (31 cases) compared to the second third time periods (five and eight cases, respectively, p < 0.0001). Relapse patient records were 2.7 times more likely to report aches (p = 0.004) and 2.8 times more likely to note lymphadenopathy (p = 0.04).

**Figure 2:**
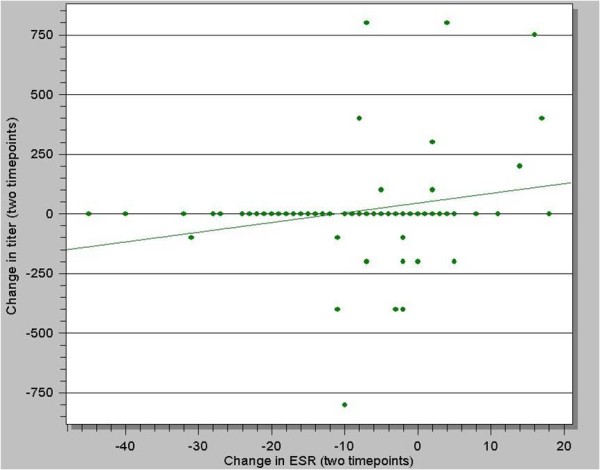
**Correlation of erythrocyte sedimentation rate (ESR) with agglutination titer in patients with brucellosis (p = 0.01)**.

Only 2.7% of patients had anemia. Leukocytosis was detected in 7.4% with 69.6% of patients with lymphocytosis. An additional 12.5% had leucopaenia. Only approximately half (64.6%) of the brucellosis patients reviewed had an elevated ESR. Interestingly, only 1.21% of chronic brucellosis cases had elevated ESR. There was no association between ESR and complications. Rising agglutination titer was not associated with rising leukocyte count, while increasing titer correlated with increasing ESR (p = 0.01) (figure 2). On the other hand, change in ESR was not associated with fever, complicated disease or vaccine therapy.

Routine antibiotic treatment at IPTM was three courses of different antibiotics with 10 days of treatment interruption between each course. The typical regimen was to start with tetracycline, continue with chloramphenicol, followed by doxycycline. The long term administration of antibiotics was generally avoided because it was regarded as harmful and possibly leading to the development of fungal infections, specifically candidiasis. A total of 19.1% of patients received antibiotic treatment before admitting to the IPTM. The most commonly used antibiotic was chloramphenicol.

### Vaccine therapy

Vaccine therapy was applied to 50 patients (17.1%), primarily in the first time period (1970-73). The majority of patients that received vaccine therapy were those diagnosed with acute brucellosis (26/50 vaccinated patients). A greater proportion of relapse patients received vaccine therapy (15/44, 34%) than those with a final diagnosis of acute (26/157, 16.6%), latent (9/63, 14.3%), or status post-brucellosis (0/10). Most of the patients with relapse were treated by the vaccine and no antibiotics. One course of vaccine therapy typically included 15 injections. Each administration of vaccine typically induced a temperature reaction of more than 38°C. In 22.9% of patients receiving vaccine therapy, the treatment was discontinued due to side effects, which included nausea, vomiting, temperature >40°C, and hematuria. An additional 8.3% of patients receiving vaccine therapy requested that the treatment cease.

**Figure 3:**
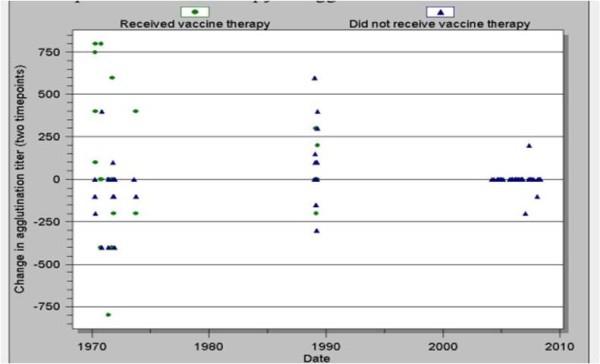
**Impact of vaccine therapy on agglutination titer by decade (p = 0.031)**.

The vaccine treatment group were 4.3 times as likely (p = 0.031) to have a rising agglutination titer between the first and second blood draws (vaccination occurred between the two blood draws). This association remained when adjusting for age and decade. The average change in titer for patients that received vaccine therapy was twofold, whereas on average, non-vaccinated patients did not demonstrate an increase in agglutination titer between the first and second blood draw (figure 3).

## Conclusions

Brucellosis continues to have a substantial impact on public health in many countries of the world, with particular importance in Georgia. To increase the understanding of this complex infection and improve worldwide control, it is useful to review the past experience of different countries as well as examine trends in epidemiology and clinical manifestations over time [18,19]. At the IPTM, a number of interesting trends were observed, including substantial shifts in the demographics of brucellosis cases, clinical differences between adult and pediatric cases, interesting patterns of initial clinical symptoms and laboratory findings, and a substantial increase in complications on presentation. The possible explanations for these trends are discussed below. Additionally, vaccine therapy was documented to have an immunogenic response, evidenced by rising agglutination titers, without a therapeutic effect.

Azeri ethnicity comprised an increasing proportion of cases over time. In the third time period, cases of brucellosis in Azeris were twice as much as cases in ethnic Georgians. There are many possible explanations for this observation. One possible contributing factor is that the Azeri population in Georgia has increased from 5.1% in 1979 to 6.5% according to the last census in 2002 [20]. Another possible explanation is that Azeris in Georgia tend to be employed at occupations at high-risk for brucellosis, such as sheep herding. Health care utilization may also explain these observations, as Azeri patients may be more prone to stay in the hospital, while Georgians prefer out-patient treatment, in which case, medical charts are not maintained. The predominance of males over females may be a result of activities that males primarily perform that lead to exposure to the infection: working as shepherds, slaughtering and skinning animals, processing animal hides, etc. There was also an observed trend of more cases in children in the recent period. This observation may be related to increasing involvement of children in the farm activities, because of economic constraints in the country. Sometimes, children are taken to the pastures as the families follow their sheep herds.

Some of the interesting aspects of the clinical observations from this review are worth mentioning. The high percentage of children with osteoarticular complications is a finding to emphasize to practitioners in the region, and to alert pediatric specialists in endemic areas. Prior studies have described osteoarticular findings in children [12,21]. In our survey, children were more likely to have these complications, thus providing data to the medical community of the need for vigilance for these findings in children. Another prior observation in children is the frequency of cytopenias, including pancytopenia, associated with brucellosis [22]. We observed a low rate of anemia (2.7%), but a significant percentage of patients with leucopenia (12.5%). It is likely the higher percentages of pancytopenia described in the previous report was attributable to a higher number of pediatric patients in that study. Our study adds further evidence to the observation that leucopenia can be associated with brucellosis.

Only approximately half of the brucellosis patients had an elevated erythrocyte sedimentation rate. This observation is surprising in the setting of an infection that should produce inflammation. However, it has been reported that chronic brucellosis patients may not have fever present [14,16], suggesting an adaptive response to the chronic presence of *Brucella *organisms in the body. This observation may suggest that the ability of *Brucella *species to evade the immune system occurs in patients, especially with chronic infection, thus leading to a disproportionately low level of inflammation. Prospective studies should investigate the pattern of inflammatory markers in brucellosis patient over time, thus providing further insight into the interaction between the pathogen and the immune system.

Neuropsychiatric symptoms were rarely reported in the documentation of the initial presentation of brucellosis, even though many patients had a prolonged duration of infection on presentation. Brucellosis has been documented to cause neuropsychiatric symptoms, although the true incidence of these symptoms associated with active infection is unknown, but likely underreported. It is likely that the brucellosis patients studied were not evaluated for these symptoms, due to the low clinical awareness of the potential neurophychiatric impact of brucellosis [23]. This practice still occurs in Georgia and physicians in other countries may follow a similar practice pattern. It is also interesting that this finding did not change over the three decades of observation. Prospective assessment of neuropsychiatric findings with brucellosis is needed, and these findings could be utilized in campaigns to educate physicians on this effect of infection.

The proportion of patient records with documented complications increased over time, especially osteoarticular complications. This trend possibly reflects the improved clinical recognition and diagnosis of complications, rather than a real increase in complications. Alternatively, this trend may indicate changing patterns of access to medical care. The actual cause of complications with brucellosis is unknown, but host susceptibility factors, bacterial strain, route of exposure, and delayed treatment may all contribute. The duration of infection before treatment may have changed over time, as decreasing access to health care may have led to increased rates of complications. Neither the agglutination titer nor the ESR was elevated in complicated disease, leading to the conclusion that these tests were not helpful in predicting complicated infections. These observations indicate that clinicians in referral centers similar to IPTM should carefully evaluate all potential brucellosis cases for the complications described in this study.

The occurrence of relapsed brucellosis infection after initial treatment was most common during 1970-1973, which was also the period of widespread use of exclusive vaccine therapy. It is also possible that in recent years, patients with relapse more often chose out-patient treatment. Therefore, relapsed cases would not be observed as frequently in the inpatient population, from which the chart review was conducted.

Vaccine therapy is a treatment method that was used exclusively in countries of the former Soviet Union. Soviet scientists tried different routes of vaccine administration: subcutaneous, intradermal, intramuscular, and intravenous [24]. In Georgia, intravenous use of vaccine was practiced [14]. In our study, the vaccine therapy was related to the increased agglutination titer, which means the activation of humoral immunity. Presently, vaccine therapy is not utilized because of its ineffectiveness, discomfort, and adverse events to the patient, as well as antibiotics being a more effective treatment option.

The limitations of the study were primarily associated with challenges inherent to retrospective review of clinical and epidemiologic information. First, our analysis does not fully reflect the actual morbidity with brucellosis in Georgia, as not all brucellosis cases in the country are referred to IPTM. Also, a referral bias could affect our conclusions as only physicians from certain regions or only certain types of patients would be referred to IPTM. Second, we could only review hospitalized cases where charts are available. There is no documentation for outpatient cases except for the registration log. Utilization of inpatient cases may introduce a bias in favor of more difficult or complicated cases, which would explain the high rate of complications detected in the more recent time periods. The most recent time period may be susceptible to bias, in which cost of hospitalization may be difficult for patients to pay, thereby selecting a unique population. As mentioned above, charts were occasionally missing epidemiological information. Additionally, out of the three courses of the antibiotic treatment, only the first treatment course was recorded from the inpatient record, as subsequent treatments were recorded in outpatient records. The limited diagnostic capacity in earlier decades may have prevented the accurate detection of complications.

The presented chart review provides substantial initial information that public health and medical personnel can utilize to improve health in Georgia. Education campaigns about brucellosis to health care providers in Georgia can lead to better recognition of the clinical and laboratory manifestations and complications of infection. Pediatricians, in particular, may benefit from discussions of the contrasting presentations of brucellosis between adults and children; namely, higher temperature on presentation and more common osteoarticular complications. Further epidemiologic investigation will be recommended for populations that referred high numbers of cases. This review provides a baseline measurement for comparison as further changes in health care and management of brucellosis occur in Georgia. Finally, reviewing the experience of vaccine therapy provides useful information for the *Brucella *vaccine development, providing documentation of immunogenic effects but ineffectiveness as a therapy. Our data suggest human *Brucella *vaccine candidates need to utilize correlates of protection that are based on the cell-mediated immune response, as the vaccine caused an increase in serological measurements, but was observed to be ineffective.

## Competing interests

The authors declare that they have no competing interests.

## Authors' contributions

All authors contributed to the development of the project concept, development of hypotheses, study design and implementation.

All authors have read and approved the final version of the manuscript.

TA abstracted the patient charts, performed initial data analysis and was the primary author for the manuscript. DVC contributed with database creation, data analysis and interpretation, and manuscript writing. GC and OZ provided local experts's opinion during the questionnaire creation, advised on the selection of timelines, and reviewed the manuscript. MJH contributed with project supervision, data analysis, manuscript writing, and final approval of manuscript content.

## Pre-publication history

The pre-publication history for this paper can be accessed here:

http://www.biomedcentral.com/1471-2334/10/346/prepub
